# Risk factors and a nomogram for predicting valproic acid-induced liver injury: A nested case-control study

**DOI:** 10.17305/bb.2024.11258

**Published:** 2024-11-29

**Authors:** Yue Chen, Yadong Wang, Runan Xia, Yi Chen, Xuefeng Xie

**Affiliations:** 1 School of Pharmacy, Anhui Medical University, Hefei, China; 2 Department of Pharmacy, Lu’an People’s Hospital, Lu’an, China; 3 Inflammation and Immune Mediated Diseases Laboratory of Anhui Province, Anhui Medical University, Hefei, China; 4 Anhui Provincial Drug Regulatory Scientific Research Center, Anhui Medical University, Hefei, China; 5 The First School of Clinical Medicine, Anhui Medical University, Hefei, China

**Keywords:** Valproic acid, VPA, liver injury, prescription sequence analysis, PSA, nested case-control, predictive model

## Abstract

The risk factors for liver injury induced by valproic acid (VPA) are not well understood, and no predictive tool currently exists to identify patients at risk. This study aims to explore these risk factors and develop a predictive model. We collected medical data from patients treated with VPA between January 1, 2020, and October 31, 2023. Prescription sequence analysis was used to identify patients with suspected VPA-induced liver injury, and the Roussel Uclaf Causality Assessment Method was applied to confirm the diagnosis. Risk factors were analyzed using logistic regression, and a nomogram model was developed and evaluated. A total of 256 cases were included in the study: 64 in the VPA-induced liver injury group and 192 in the control group. The incidence of liver injury was 5.3%. Multivariate logistic regression analysis revealed that dysglycemia (odds ratio [OR] ═ 5.171; 95% confidence interval [CI]: 1.254–21.325), hyperlipidemia (OR ═ 4.903; 95% CI: 1.400–17.173), surgery (OR ═ 10.020; 95% CI: 1.737–57.805), and hypokalemia (OR ═ 10.407; 95% CI: 2.398–45.173) were significant independent risk factors for VPA-induced liver injury. The area under the receiver operating characteristic curve was 0.904 (95% CI: 0.860–0.947), indicating excellent model performance. The Hosmer–Lemeshow test yielded a *P* value of 0.2671, and the calibration plot slope was close to one, further supporting the model’s accuracy. The findings suggest that patients with dysglycemia, hyperlipidemia, a history of surgery, and hypokalemia are at higher risk for VPA-induced liver injury. The nomogram model provides a reliable method for predicting the likelihood of liver injury in these patients.

## Introduction

Drug-induced liver injury (DILI) has become the leading cause of acute liver failure worldwide, with its incidence steadily rising. In China, the estimated annual incidence of DILI is at least 0.0238%, which is notably higher than in other countries and continues to increase yearly. Among hospitalized patients, the incidence of DILI is approximately 1%–6% [1].

Valproic acid (VPA), a first-line antiepileptic drug with a broad spectrum of efficacy, is often used in combination with other antiepileptic drugs, such as lamotrigine, levetiracetam, and phenytoin sodium. Beyond epilepsy, VPA is also widely prescribed for migraine, mood disorders, anxiety, and bipolar disorder [2]. However, with its growing clinical use, VPA has been associated with numerous adverse drug reactions, including hemorrhagic pancreatitis, bone marrow suppression, obesity, teratogenicity, and liver injury [3, 4]. Among these, liver injury is one of the most severe complications.

VPA undergoes extensive hepatic metabolism via a highly complex metabolic pathway [5]. Food and Drug Administration (FDA) has warned of the potential for serious or life-threatening liver damage associated with VPA [6]. Additionally, patients treated with VPA may exhibit signs of fatty liver and steatosis on ultrasound [7]. Acute liver injury, a severe adverse reaction, often occurs within the first six months of treatment. Clinical manifestations include nausea, vomiting, hepatocellular necrosis, cholestatic liver injury, or elevated serum transaminase levels, with severe cases potentially progressing to acute liver failure. The mortality rate of acute liver failure remains high, and there are currently no specific therapeutic agents for its treatment [8].

Despite the risks, the incidence, clinical characteristics, and risk factors for VPA-induced liver injury remain poorly understood, and no predictive tools currently exist. Identifying high-risk patients in advance and implementing preventive measures could significantly reduce the risk of VPA-induced liver injury. However, existing clinical studies on this issue primarily focus on gene polymorphisms and plasma drug concentrations [9–11]. While these studies have provided preliminary insights into the condition, research on its risk factors and predictive models remains scarce.

**Figure 1.**
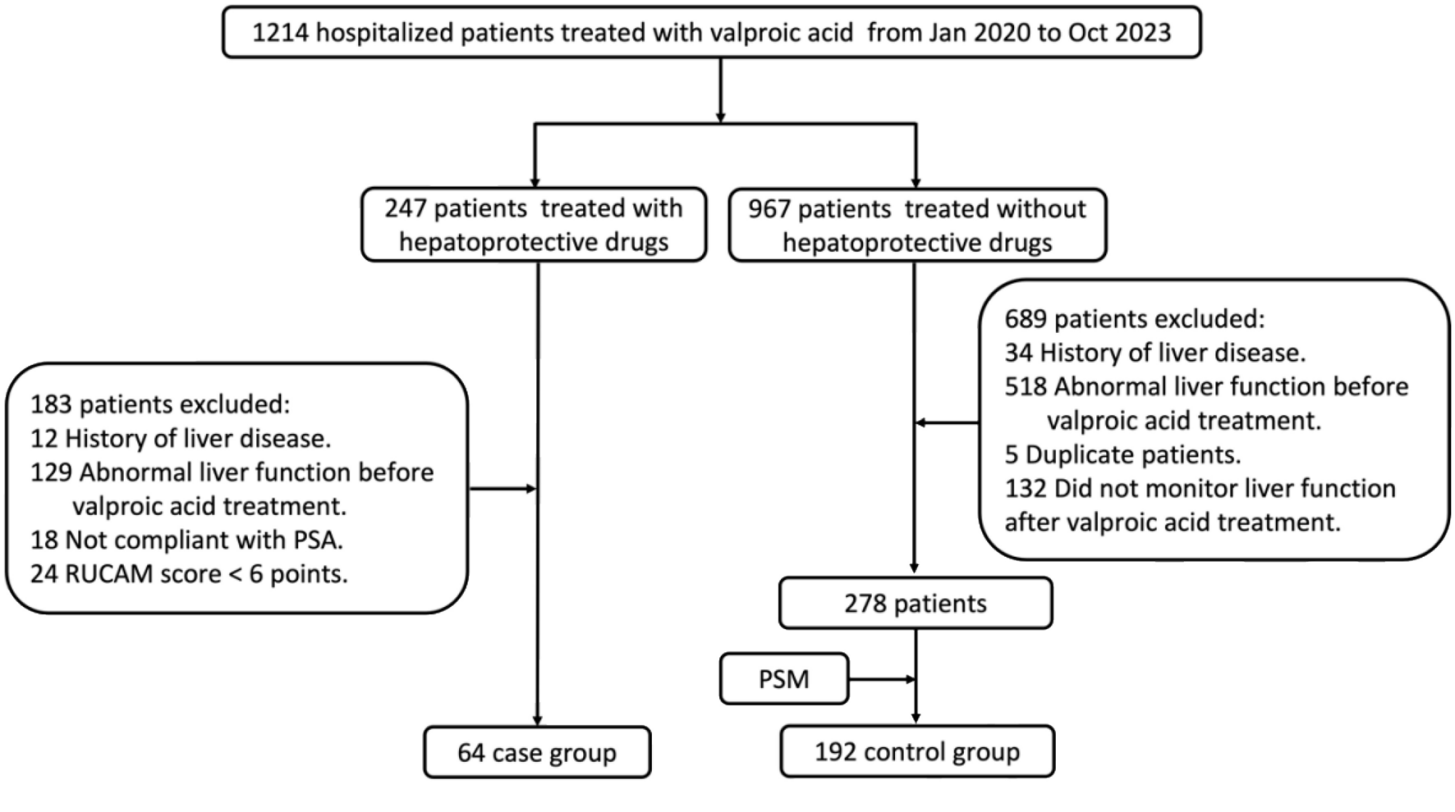
**Flowchart of case selection and identification.** PSA: Prescription sequence analysis; PSM: Propensity score matching; RUCAM: Roussel Uclaf Causality Assessment Method.

In recent years, nomogram models have gained prominence in medical research for predicting, diagnosing, and assessing disease prognosis. These models visually represent complex statistical data, offering advantages such as intuitiveness, accuracy, and practicality, thus supporting clinical decision-making. This study aimed to investigate the incidence and risk factors of VPA-induced liver injury in real-world settings. Furthermore, it developed a predictive nomogram model to identify high-risk patients, enabling timely intervention and appropriate management. Ultimately, these efforts aim to enhance the safety profile of VPA administration.

## Materials and methods

### Research design and data sources

We conducted a nested case-control study (NCCS) at a tertiary hospital in Anhui Province. A cluster sampling method was employed to include all hospitalized patients who received VPA injections between January 1, 2020, and October 31, 2023. To maintain data quality, all investigators underwent unified and standardized training prior to the study.

### Case selection and identification

Patients meeting the following inclusion criteria were considered for the study: (1) hospitalized patients treated with VPA and (2) patients with normal liver function prior to the initiation of VPA treatment. The exclusion criteria were: (1) patients receiving VPA for less than 24 h, (2) patients with preexisting liver-related conditions, such as hepatitis, fatty liver, or liver malignancy, and (3) patients with incomplete medical records. Liver function was assessed using the following normal reference ranges: total bilirubin (TBIL) 2–17 µmol/L, alanine aminotransferase (ALT) 0–40 U/L, and aspartate aminotransferase (AST) 0–40 U/L. The criteria for liver injury: ALT > 2 times the upper limit of normal (2×ULN) and/or AST > 2×ULN, and/or TBIL> 2×ULN. Prescription sequence analysis (PSA) was employed to identify suspected cases of VPA-induced liver injury. Specifically, suspected cases were those in which hepatoprotective medications—such as magnesium isoglycyrrhizinate, diammonium glycyrrhizinate, glutathione, or liver-protecting tablets—were administered following the initiation of VPA treatment. Two clinical pharmacists independently evaluated these suspected cases using the Roussel Uclaf Causality Assessment Method (RUCAM) [12]. Cases with a total RUCAM score ≥ 6 (indicating a “very likely” causality) were classified as VPA-induced liver injury and included in the case group.

Propensity score matching (PSM) was then used to match patients in the case group with those who maintained normal liver function and did not require hepatoprotective drugs during VPA treatment. Matching was based on sex and age. Once a control patient was successfully matched to a case, the control was no longer eligible for matching with other cases. The case-to-control matching ratio was 1:3. A detailed flowchart illustrating case selection and identification is presented in Figure 1.

### Data collection

The medical information of the patients was collected, including demographic and clinical details, such as sex, age, single-dose administration of VPA, treatment duration, and nutritional risk screening (NRS) score. Laboratory parameters were recorded, including ALT, AST, TBIL, total protein (TP), albumin (ALB), creatinine (Cr), and international normalized ratio (INR) prior to VPA injection. Data on concurrent medication use were gathered, including the combination of antiepileptic drugs (defined as being treated with two or more antiepileptic drugs simultaneously during hospitalization). Additionally, the history of allergies (e.g., allergic reactions to food, drugs, or substances like pollen), smoking history (defined as continuous or cumulative smoking for six months or more in a lifetime), and drinking history (for men, an average daily alcohol intake exceeding 40 g; for women, exceeding 20 g; drinking time ≥5 years; or short-term heavy alcohol consumption, defined as an average daily intake exceeding 80 g for two weeks) was assessed. The history of malignant tumors (having had or currently suffering from a malignant tumor) was recorded, as well as the presence of concurrent diseases, such as hyperlipidemia (evidenced by one or more of the following: elevated triglycerides, elevated total cholesterol, elevated low-density lipoprotein cholesterol, or decreased high-density lipoprotein cholesterol), hypertension, cardiopathy (including coronary heart disease, myocardial infarction, heart failure, and arrhythmia), and cerebral infarction. The presence of infections (bacterial or viral) during hospitalization was noted, along with electrolyte imbalances, such as hypokalemia and hyponatremia, and other conditions, including anemia, dysglycemia (diabetes or stress hyperglycemia), and other symptoms observed before VPA treatment. Information was also gathered on whether the patients underwent surgery before or during VPA treatment and whether they received parenteral nutrition (PN) during the same period.

### Model performance evaluation

To evaluate the model’s ability to identify patients with VPA-induced liver injury, a receiver operating characteristic (ROC) curve was generated using data from the multivariate logistic regression analysis. The area under the curve (AUC) was calculated along with its 95% confidence interval (CI). To assess the model’s overall performance and goodness-of-fit, the omnibus Hosmer–Lemeshow test was employed. Additionally, a calibration plot was created to compare the observed probabilities with the model-predicted probabilities, thereby assessing the model’s calibration accuracy.

### Ethical statement

This study was performed in accordance with the principles of the Declaration of Helsinki. Approval was granted by the Biomedical Ethics Committee of Anhui Medical University (No: 83244648). The need for individual consent for this study was waived.

### Statistical analysis

Microsoft Excel 2021 was used for data organization, SPSS (version 26.0) for statistical analysis, and RStudio (version 4.3.2) for PSM as well as the development and evaluation of the predictive model. The one-sample Kolmogorov–Smirnov test was used to assess the normality of continuous variables. Normally distributed variables were presented as mean ± standard deviation and compared using Student’s *t*-test. Non-normally distributed variables were presented as medians (25th and 75th percentiles) and compared using the Mann–Whitney *U* test. Categorical variables were reported as frequencies and proportions, with comparisons conducted using chi-square or Fisher’s exact tests. In the predictive model, liver injury was the dependent variable. Key exposure factors included hyperlipidemia, a history of allergies, combination of antiepileptic drugs, age ≥ 60 years, NRS score ≥ 3 points, hypertension, cerebral infarction, cardiopathy, dysglycemia, smoking history, drinking history, malignant tumors, concurrent infections, anemia, treatment course > 7 days, surgery, concurrent use of PN, hypokalemia, and hyponatremia. Univariate logistic regression was first performed to analyze these variables individually. Statistically significant factors were then included in a multivariate logistic regression analysis. As the proportion of missing data was minimal, cases with missing data were excluded from all analyses. The *P* values, odds ratios (ORs), and 95% CIs for the associations between variables and the risk of VPA-induced liver injury were calculated. *P* < 0.05 was considered to indicate statistical significance.

## Results

### Basic information of the case group and the control group

From January 1, 2020, to October 31, 2023, a total of 1214 hospitalized patients were treated with VPA. After applying inclusion and exclusion criteria, 64 patients were included in the case group. Using sex and age as matching conditions, 192 patients were successfully matched via the PSM method and included in the control group. The results showed that the incidence of VPA-induced liver injury was 5.3%. Baseline information is presented in Table 1.

**Table 1:** Baseline information of the valproic acid-induced liver injury group and control group

**Characteristics**	**Valproic acid-induced liver injury group**	**Control group**	***P* value**
Number (*n*)	64	192	
Age (mean ± SD, years)	58.3 8 ± 11.43	61.81 ± 13.05	0.062
TBIL (mean ± SD, µmol/L)	11.58 ± 3.29	11.47 ± 3.23	0.803
TP (mean ± SD, g/L)	59.70 ± 10.80	63.14 ± 7.77	0.021*
ALB (mean ± SD, g/L)	39.17 ± 8.14	39.36 ± 6.17	0.869
AST (mean ± SD, IU/L)	24.42 ± 8.26	21.03 ± 6.82	0.004**
ALT (mean ± SD, IU/L)	18.03 ± 7.29	15.99 ± 7.65	0.063
Treatment course (M(P_25_,P_75_), days)	8.00 (5.00, 12.00)	4.00 (3.00, 7.00)	0.000***
Cr (M(P_25_,P_75_), µmol/L)	60.25 (49.20, 75.70)	63.85 (51.83, 80.43)	0.210
INR (M(P_25_,P_75_))	1.01 (0.97, 1.08)	0.99 (0.93, 1.06)	0.051
Total dose (M(P_25_,P_75_), g)	9.60 (6.40, 15.60)	6.00 (3.60, 8.40)	0.000***
*Number of antiepileptic drugs, n (%)*			
1	13 (20.3)	94 (49.0)	0.000***
2	24 (37.5)	68 (35.4)	
3	27 (42.2)	30 (15.6)	
*Sex, n (%)*			
Male	45 (70.3)	135 (70.3)	/
Female	19 (29.7)	57 (29.7)	
*Hyperlipidaemia, n (%)*			
Yes	44 (68.8)	57 (29.7)	0.000***
No	20 (31.3)	135 (70.3)	
*History of allergies, n (%)*			
Yes	3 (4.7)	7 (3.6)	0.710
No	61 (95.3)	185 (96.4)	
*Hypertension, n (%)*			
Yes	44 (68.8)	116 (60.4)	0.233
No	20 (31.3)	76 (39.6)	
*Cerebral infarction, n (%)*			
Yes	27 (42.2)	93 (48.4)	0.386
No	37 (57.8)	99 (51.6)	
*Cardiopathy, n (%)*			
Yes	4 (6.3)	26 (13.5)	0.116
No	60 (93.8)	166 (86.5)	
*Dysglycemia^a^, n (%)*			
Yes	50 (78.1)	69 (35.9)	0.000***
No	14 (21.9)	123 (64.1)	
*Smoking history, n (%)*			
Yes	7 (10.9)	17 (8.9)	0.620
No	57 (89.1)	175 (91.1)	
*Drinking history, n (%)*			
Yes	8 (12.5)	15 (7.8)	0.256
No	56 (87.5)	177 (92.2)	
*Malignant tumor, n (%)*			
Yes	3 (4.7)	8 (4.2)	0.859
No	61 (95.3)	184 (95.8)	
*Concurrent infection, n (%)*			
Yes	63 (98.4)	135 (70.3)	0.000***
No	1 (1.6)	57 (29.7)	
*Anemia, n (%)*			
Yes	14 (21.9)	21 (10.9)	0.027*
No	50 (78.1)	171 (89.1)	
*Surgery, n (%)*			
Yes	60 (93.8)	94 (49.0)	0.000***
No	4 (6.3)	98 (51.0)	
*Combined with PN, n (%)*			
Yes	26 (40.6)	45 (23.4)	0.008**
No	38 (59.4)	147 (76.6)	
*Hypokalemia, n (%)*			
Yes	50 (78.1)	65 (33.9)	0.000***
No	14 (21.9)	127 (66.1)	
*Hyponatremia, n (%)*			
Yes	44 (68.8)	75 (39.1)	0.000***
No	20 (31.3)	117 (60.9)	
*NRS score, n (%)*			
0	5 (7.8)	43 (22.4)	0.001**
1	1 (1.6)	17 (8.9)	
2	13 (20.3)	25 (13.0)	
3	19 (29.7)	64 (33.3)	
4	10 (15.6)	23 (12.0)	
5	13 (20.3)	12 (6.3)	
6	2 (3.1)	7 (3.6)	
7	1 (1.6)	1 (0.5)	
*Daily dose (g), n (%)*			
0.4	0 (0)	1 (0.5)	0.332
0.8	0 (0)	10 (5.2)	
1.0	1 (1.6)	1 (0.5)	
1.2	50 (78.1)	142 (74.0)	
1.44	0 (0)	1 (0.5)	
1.6	13 (20.3)	37 (19.3)	

^a^Including diabetes and stress hyperglycemia. Categorical variables are shown as numbers and percentages. Continuous variables with normal distribution are expressed as mean ± SD. Continuous variables with a nonnormal distribution are expressed as M(P_25_,P_75_). *P < 0.05. **P < 0.01. *** *P* < 0.001. SD: Standard deviation; TBIL: Total bilirubin; TP: Total protein; ALB: Albumin; AST: Aspartate aminotransferase; ALT: Alanine aminotransferase; M(P_25_,P_75_): Median (25^th^, 75^th^ percentiles); INR: International normalized ratio; PN: Parenteral nutrition; NRS: Nutritional risk screening.

Among the participants, there were 180 males (45 in the case group and 135 in the control group) and 76 females (19 in the case group and 57 in the control group). The average age of the patients in the case group was 58.38 ± 11.43 years, compared to 61.81 ± 13.05 years in the control group. The age difference between the two groups was not statistically significant (*P* > 0.05).

A comparison of the characteristics of patients with and without liver injury revealed no significant differences in TBIL, ALB, ALT, Cr, INR, or the presence of allergy history, hypertension, cerebral infarction, cardiopathy, smoking history, drinking history, malignant tumor status, or daily dose of VPA. However, compared to the control group, patients in the case group exhibited lower TP, higher AST, longer treatment courses, greater total doses, a greater number of anti-epileptic drug types, and higher rates of hyperlipidemia, dysglycemia, surgery, anemia, concurrent infections, PN use, hypokalemia, and hyponatremia.

### Univariate logistic regression analysis of risk factors for VPA-induced liver injury

Using liver injury as the dependent variable, exposure factors were analyzed through univariate logistic regression. The results indicated that hyperlipidemia, combination of antiepileptic drugs, age ≥ 60 years, an NRS score ≥ 3 points, dysglycemia, concurrent infection, anemia, a treatment course > 7 days, surgery, combination with PN, hypokalemia, and hyponatremia were all significantly associated with VPA-induced liver injury (*P* < 0.05) (Table 2).

**Table 2:** Univariate logistic regression analysis of risk factors for valproic acid-induced liver injury

**Factor**	**β**	**SE**	**Wald**	***P* value**	**OR**	**95% CI**	
Hyperlipidaemia	1.569	0.326	23.147	0.000***	4.803	2.534	9.101
History of allergies	0.271	0.718	0.142	0.706	1.311	0.321	5.357
Combination of antiepileptic drugs	1.439	0.370	15.127	0.000***	4.218	2.042	8.713
Age ≥ 60 years	−2.563	1.036	6.116	0.013*	0.077	0.010	0.588
NRS score ≥ 3 points	0.651	0.319	4.167	0.041*	1.918	1.026	3.584
Hypertension	0.438	0.341	1.644	0.200	1.549	0.793	3.025
Cerebral infarction	−0.258	0.294	0.772	0.380	0.772	0.434	1.375
Cardiopathy	−0.817	0.549	2.217	0.136	0.442	0.151	1.295
Dysglycemia^a^	1.958	0.378	26.856	0.000***	7.082	3.378	14.848
Smoking history	0.248	0.490	0.257	0.612	1.282	0.491	3.348
Drinking history	0.543	0.476	1.300	0.254	1.722	0.677	4.381
Malignant tumor	0.126	0.700	0.032	0.858	1.134	0.288	4.472
Concurrent infection	3.342	1.024	10.655	0.001**	28.270	3.801	210.263
Anemia	0.822	0.382	4.623	0.032*	2.274	1.075	4.811
Treatment course > 7 days	1.440	0.320	20.217	0.000***	4.220	2.253	7.906
Surgery	2.702	0.533	25.742	0.000***	14.915	5.251	42.365
Combined with PN	0.783	0.305	6.602	0.010*	2.189	1.204	3.978
Hypokalemia	1.706	0.324	27.690	0.000***	5.505	2.916	10.391
Hyponatremia	1.252	0.319	15.410	0.000***	3.498	1.872	6.536

^a^Including diabetes and stress hyperglycemia. *P < 0.05. **P < 0.01. *** *P* < 0.001. SE: Standard error; OR: Odds ratio; CI: Confidence interval; NRS: Nutritional risk screening; PN: Parenteral nutrition.

### Multivariate logistic regression analysis of risk factors for VPA-induced liver injury

The variables with significant differences in Table 2 were included in the equation for the multivariate logistic regression analysis. The results indicated that dysglycemia (OR ═ 5.171; 95% CI: 1.254–21.325), hyperlipidemia (OR ═ 4.903; 95% CI: 1.400–17.173), surgery (OR ═ 10.020; 95% CI: 1.737–57.805), and hypokalemia (OR ═ 10.407; 95% CI: 2.398–45.173) were statistically significant independent risk factors (*P* < 0.05) (Table 3).

**Table 3:** Multivariate logistic regression analysis of risk factors for valproic acid-induced liver injury

**Factor**	**β**	**SE**	**Wald**	***P* value**	**OR**	**95% CI**	
Dysglycemia^a^	1.643	0.723	5.165	0.023*	5.171	1.254	21.325
Hyperlipidaemia	1.590	0.640	6.180	0.013*	4.903	1.400	17.173
Combination of antiepileptic drugs	0.733	0.694	1.118	0.290	2.082	0.535	8.109
Age ≥ 60 years	−1.799	1.203	2.234	0.135	0.166	0.016	1.751
NRS score ≥ 3 points	0.307	0.628	0.239	0.625	1.360	0.397	4.660
Concurrent infection	1.371	1.354	1.026	0.311	3.940	0.278	55.923
Anemia	1.532	0.911	2.829	0.093	4.626	0.776	27.574
Treatment course > 7 days	−0.034	0.590	0.003	0.954	0.967	0.304	3.074
Surgery	2.305	0.894	6.643	0.010*	10.020	1.737	57.805
Combined with PN	−0.061	0.629	0.009	0.922	0.941	0.274	3.224
Hypokalemia	2.343	0.749	9.782	0.002**	10.407	2.398	45.173
Hyponatremia	0.334	0.624	0.287	0.592	1.397	0.412	4.741

^a^Including diabetes and stress hyperglycemia. *P < 0.05. **P < 0.01. *** *P* < 0.001. SE: Standard error; OR: Odds ratio; CI: Confidence interval; NRS: Nutritional risk screening; PN: Parenteral nutrition.

### Development and evaluation of the nomogram model for VPA-induced liver injury

**Figure 2.**
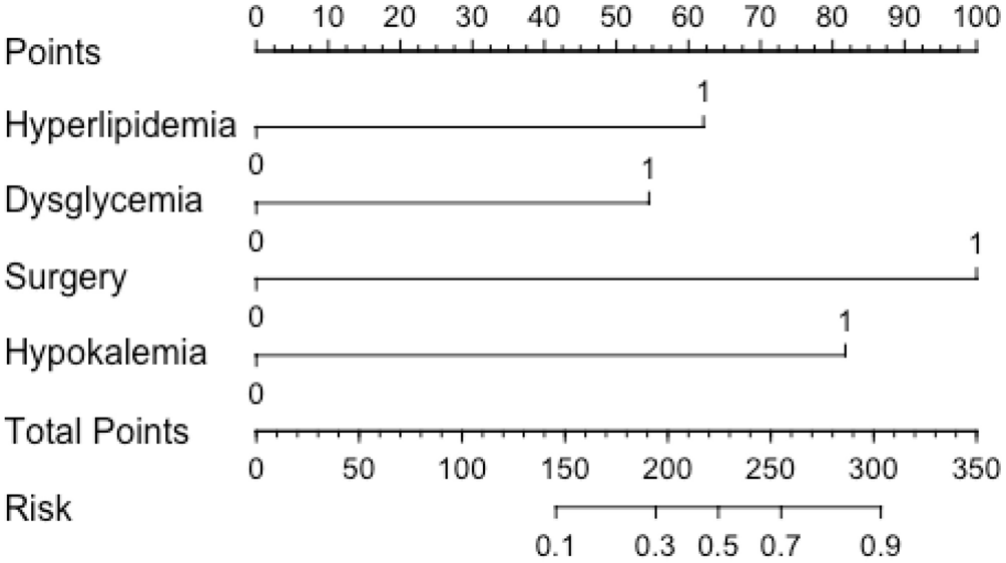
A nomogram of risk factors for valproic acid-induced liver injury.

**Figure 3.**
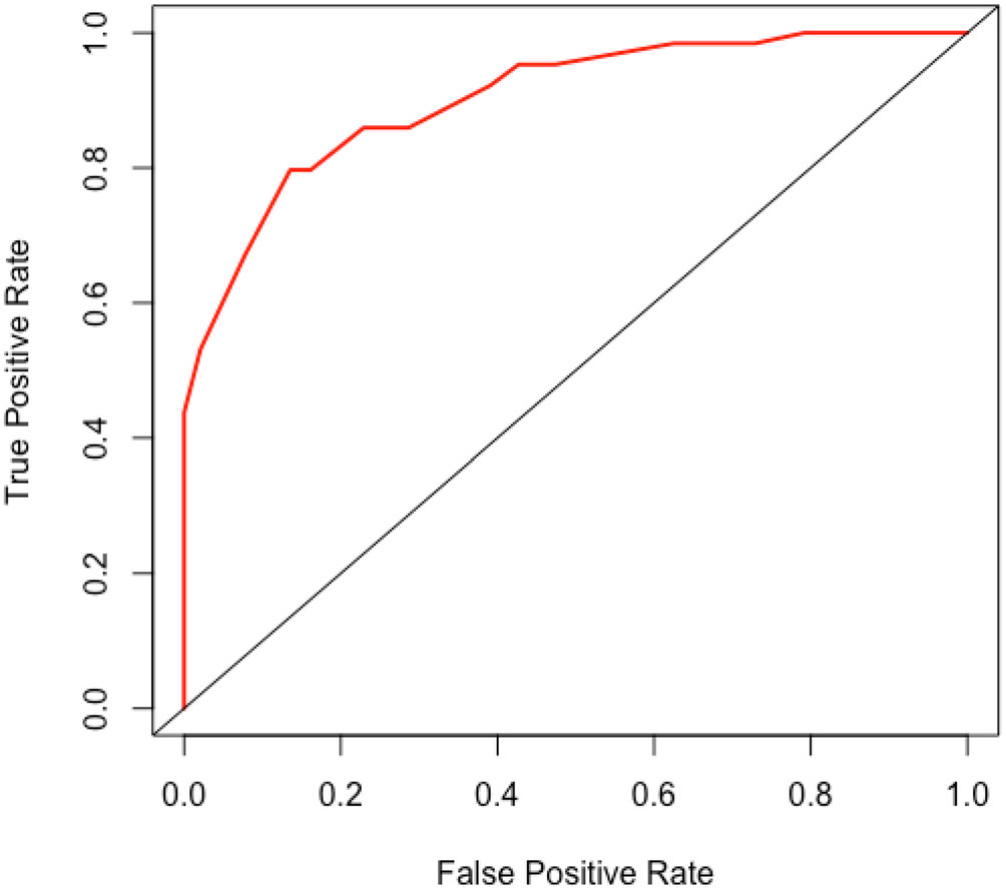
Receiver operating characteristic (ROC) curve of the model.

**Figure 4.**
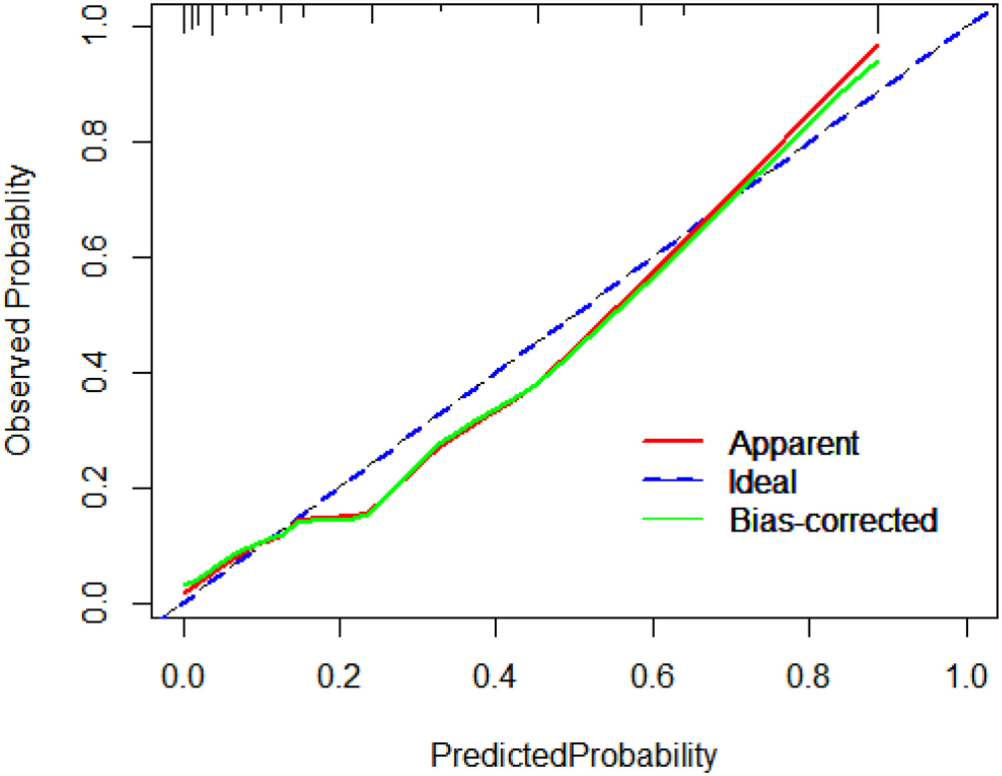
Calibration plot of the model.

The variables of the predictive model were determined through multivariate logistic regression analysis. The model was developed using the “rms” package in RStudio (version 4.3.2), and the “nomogram” function in the package was employed to generate a nomogram. As illustrated in Figure 2, the model score for patients with dysglycemia increased by 54 points compared to those with normal blood sugar. Similarly, patients with hyperlipidemia had a model score 62 points higher than those without hyperlipidemia, while patients with hypokalemia saw an 82-point increase compared to those without hypokalemia. Additionally, patients who underwent surgery experienced a 100-point increase in their model score relative to those who did not undergo surgery. The total score was then used to predict the likelihood of liver injury in patients receiving VPA treatment: the higher the score, the greater the risk of liver injury. For instance, a patient who has undergone surgery and presents with dysglycemia, hyperlipidemia, and hypokalemia would have a model score of 298 points, corresponding to an 89% probability of liver injury following VPA treatment. To assess the accuracy of the nomogram model in predicting the risk of VAP-induced liver injury, an ROC curve was plotted, as shown in Figure 3. The calculated AUC was 0.904 (CI: 0.860–0.947), demonstrating the model’s strong predictive performance. Furthermore, the model was calibrated, and a calibration plot was generated. As displayed in Figure 4, the slope of the calibration plot was close to one, indicating a high degree of agreement between the predicted and actual risk of liver injury. The Hosmer–Lemeshow test result was *P* ═ 0.2671, suggesting that the patients’ medical data were well-extracted and that the model demonstrated a good fit.

## Discussion

A PSA combined with an NCCS was utilized in this research. PSA is a research method based on an existing and complete prescription record database [13]. This method is particularly useful when an adverse reaction to one drug serves as an indication for administering another drug. Although the hospital information system does not explicitly record whether a patient has liver injury, when a patient develops liver injury after receiving VPA, it becomes an indication for treatment with hepatoprotective drugs, meeting the criteria for PSA. Compared to other pharmacoepidemiological research methods, PSA is cost-effective and less time-intensive. However, as a retrospective and observational study, the presence of confounding factors and biases imposes certain limitations on the method. For instance, some patients with mild liver injury may have discontinued VPA without receiving hepatoprotective drugs. Such cases could not be identified through PSA and were therefore excluded from the study. The NCCS is a hybrid research method that combines cohort and case-control study designs. In this approach, the case group and control group are derived from the same specific cohort, ensuring more balanced and comparable data between the groups [14]. Compared to cohort studies, NCCS requires a smaller sample size, making it more efficient and cost-effective. Furthermore, unlike traditional case-control studies, the NCCS collects data on exposure factors before disease onset. This ensures a clear causal relationship and avoids time sequence ambiguity between exposure and disease onset, making it highly suitable for this study. Additionally, a nomogram model was developed to simplify the predictive model into a single numerical estimate of the probability of an event by integrating various clinical variables. In this study, the nomogram was built using four factors identified through multivariate logistic regression analysis, and its performance was subsequently evaluated. The results indicated that the nomogram model demonstrated good clinical calibration and efficiency, making it a valuable tool for clinicians to predict the probability of liver injury in patients. A total of 19 exposure factors were included in this study. To minimize the impact of bias and confounding factors on the results, strict inclusion and exclusion criteria were established. Potential risk factors for DILI include a history of drug reactions, being either very old or very young, being female (especially in cases of acute liver failure), treatment with multiple drugs metabolized by the liver within a short time, immune disorders, preexisting liver disease, poor nutritional status, and so on [15–18]. Several studies on adverse reactions in hospitalized patients have shown that age and sex are common risk factors [19, 20]. Ma and Wang [21] revealed that male patients had a greater risk of VPA-induced liver injury than female patients. In this study, cases of liver injury were identified in patients treated with VPA. Appropriate controls, who did not develop liver injury, were selected based on matching criteria, such as age and sex. By matching cases and controls from the same cohort, the study minimized the influence of confounding factors like age and sex, thereby improving the comparability between the two groups. Notably, since sex was used as a matching criterion in the PSM method, it was not analyzed as an exposure factor for the effect of sex differences on VPA-induced liver injury. This study found that dysglycemia, hyperlipidemia, hypokalemia, and surgery were associated with an increased risk of VPA-induced liver injury. The liver, as an essential organ for the metabolism of sugar, fat, and protein, plays a crucial role in mediating these effects. Verrotti et al. [22] identified insulin resistance and metabolic syndrome as predictors of VPA-induced liver injury. Patients with dysglycemia often have reduced self-regulation capacity and are more sensitive to the hepatotoxic effects of certain drugs compared to patients with normal blood sugar levels [23, 24]. This sensitivity may trigger allergic reactions in the liver, exacerbating liver injury. The increased risk of DILI in patients with hyperlipidemia may be explained by several mechanisms. First, overnutrition may elevate preexisting cellular oxidants in the host, aggravating oxidative stress in the liver, which can lead to steatosis, lipid peroxidation, and mitochondrial damage [25]. Second, patients with hyperlipidemia often receive statin therapy, and statins, which are primarily metabolized by the liver, have been shown to be hepatotoxic [26, 27]. Combining statins with other hepatotoxic drugs can heighten the liver’s vulnerability to injury. Hypokalemia also contributes to liver injury. When blood potassium levels drop, the activity of the sodium–potassium pump decreases. Muriel and Pérez-Rojas [28], in an animal study on sepsis, demonstrated that reduced activity of key adenosine triphosphatases (including the sodium–potassium pump) on the mitochondrial membrane of liver cells can inhibit mitochondrial membrane proteins. This leads to changes in membrane fluidity and permeability, disrupts energy metabolism, and ultimately results in liver injury. Surgery was also identified as a risk factor for VPA-induced liver injury. Kong et al. [29] reported that surgery increases the risk of liver injury in hospitalized patients. Surgery induces physical trauma and elicits a stress response in the body, leading to the production of cytokines, inflammatory mediators, and oxygen free radicals [30, 31]. These factors collectively heighten the susceptibility of liver cells to damage.

Several studies have demonstrated that a history of alcohol consumption increases the risk of DILI [32–34]. However, in this study, drinking history was not identified as a risk factor for VPA-induced liver injury. We speculate that this discrepancy may be influenced by variables such as alcohol consumption levels, duration of drinking history, and whether the individual had ceased drinking. Li et al. [35] found malnutrition to be a risk factor for DILI, leading us to hypothesize that nutritional status may similarly influence VPA-induced liver injury. To investigate this, we analyzed the relationship between nutritional status, as measured by NRS scores, and VPA-induced liver injury. However, our results showed no significant association. This may be because NRS scores reflect not only nutritional status but also age and disease severity. Patients receiving PN may experience severe cholestasis due to the lack of enteral feeding, with some cases resulting in fatal complications like liver injury or intestinal atrophy [36–39]. To explore whether PN increases the risk of VPA-induced liver injury, we included PN as a variable in our study. However, no association was found. We speculate this outcome may be influenced by PN treatment duration. Short-term and long-term PN may exert different effects on the body, and differences in PN types (e.g., total PN vs partial PN) may also play a role. Further rigorous studies are needed to clarify these relationships. Meseguer et al. [34] reported that the combination of antiepileptic drugs increases the risk of VPA-induced liver injury. In contrast, our findings did not identify such a combination as a risk factor. This discrepancy may stem from differences in drug regimens; for example, some patients received two antiepileptic drugs, others three, and the specific drugs combined varied. To address this, future studies should ensure consistent baseline characteristics and standardized drug combinations among patients. Guo et al. [40] identified treatment duration as a risk factor for VPA-induced liver injury, with ten days as the optimal cutoff. In our study, we used treatment durations of more than seven days as the exposure factor but found no significant association. This difference may arise because our study focused on the broader category of treatment durations exceeding seven days, rather than pinpointing specific cutoff durations. The hepatotoxicity of VPA has been linked to genetic variations in several enzymes, such as cytochrome P450, polymerase γ, glutathione S-transferases, superoxide dismutase 2, and carbamoyl phosphate synthetase 1 [5]. Additionally, two studies [21, 41] have identified the catalase C-262T genotype as a key genetic risk factor for VPA-induced liver injury. Unfortunately, genetic factors were not included in this study due to its retrospective design. Many patients did not undergo genetic testing, possibly due to economic or other constraints, preventing us from obtaining genetic data. Environmental factors were also excluded from our analysis. Retrospective studies often face challenges in controlling for such variables, as they are complex and prone to bias, which can affect results.

Lastly, the relationship between plasma concentrations of VPA and liver injury is debated. One study [40] reported an association, whereas another [42] suggested that plasma VPA concentrations in patients with abnormal liver function were not reliable predictors of adverse reactions. Instead, the ratio of 4-ene-VPA (a metabolite of VPA) to VPA was deemed a better predictor of hepatotoxicity. Regrettably, we were unable to evaluate this factor, as plasma VPA and metabolite concentrations were not routinely monitored in most cases included in this study. With this model, doctors only need to input risk factor-related data into the system before administering VPA. The model automatically calculates the risk of VPA-induced liver injury, helping to identify high-risk patients. For such patients, the treatment plan can be adjusted, or proactive measures—such as liver function monitoring—can be implemented after administering VPA to prevent liver injury. The results of the predictive model are intended solely as a reference for clinical decision-making. While they highlight patients who may be at risk of liver injury, the model does not replace the doctor’s judgment in making the final treatment decision. The strength of this study lies in its comprehensive use of patient information and medication characteristics. However, the study also has certain limitations, as it was a single-center retrospective study. Compared with large multicenter studies, this research included a relatively small sample size limited to individuals from one region. Additionally, the study population primarily consisted of middle-aged and elderly individuals, meaning it does not cover all age groups and is not broadly representative. Another limitation is the retrospective design itself, which carries the risk of data loss during collection. Although the likelihood of missing data is low, its potential impact on the overall results cannot be ignored. Moreover, retrospective studies rely on previously recorded information, which can introduce bias and affect data quality. Furthermore, the model excludes the complex effects of liver-related diseases, limiting its applicability to patients with such conditions. Therefore, careful consideration is needed when applying this model in clinical practice. To better explore the risk factors for VPA-induced liver injury, future research should focus on conducting large-scale, multicenter prospective or retrospective clinical studies to validate the clinical utility of the nomogram model developed in this study.

## Conclusion

According to the findings of this study, dysglycemia, hyperlipidemia, surgery, and hypokalemia are independent risk factors for VPA-induced liver injury. Physicians should carefully monitor these risk factors and routinely assess liver function in high-risk patients. The predictive model developed in this study shows strong potential for clinical application, enabling early screening and timely intervention in high-risk patients to enhance the safety profile of VPA therapy.

## Data Availability

The data that support the findings of this study are available from the corresponding author upon reasonable request.
